# Cancerous inhibitor of protein phosphatase 2A (CIP2A) is an independent prognostic marker in wild-type KRAS metastatic colorectal cancer after colorectal liver metastasectomy

**DOI:** 10.1186/s12885-015-1300-3

**Published:** 2015-04-17

**Authors:** Kuen-Feng Chen, Chueh-Chuan Yen, Jen-Kou Lin, Wei-Shone Chen, Shung-Haur Yang, Jeng-Kai Jiang, Yuan-Tzu Lan, Chun-Chi Lin, Hui-Chuan Yu, Hui-Mei Hsu, Wen-Ling Lin, Hao-Wei Teng

**Affiliations:** 1 Division of Hematology and Oncology, Department of Medicine, Taipei Veterans General Hospital, No. 201, Section 2, Shih-Pai Road, Taipei, Taiwan; 2 Division of Colon and Rectal Surgery, Department of Surgery, Taipei Veterans General Hospital, Taipei, Taiwan; 3 Institute of Clinical Medicine, National Yang-Ming University, Taipei, Taiwan; 4 National Yang-Ming University School of Medicine, Taipei, Taiwan; 5 Department of Medical Research, National Taiwan University Hospital, Taipei, Taiwan; 6 National Center of Excellence for Clinical Trial and Research, National Taiwan University Hospital, Taipei, Taiwan

**Keywords:** CIP2A, Colorectal neoplasm, KRAS, Liver, Metastasectomy

## Abstract

**Background:**

The impact of KRAS signaling on cancerous inhibitor of protein phosphatase 2A (CIP2A) expression has not yet been explored. We investigated the impact of KRAS on CIP2A expression in colorectal cancer patients after colorectal liver metastasectomy.

**Methods:**

We examined CIP2A expression by immunohistochemistry (IHC) and used direct sequencing to identify the mutational status of *KRAS* exon 2 (codon 12 and 13). The association between CIP2A expression, *KRAS* genotype, clinicopathological parameters and survival were examined by the Kaplan–Meier method and the Cox proportional hazards model. A combination of immunoblotting and proliferation assays were employed to elucidate the role of CIP2A in signal transduction pathways in wild-type *KRAS* Caco-2 cells.

**Results:**

A total of 220 colorectal cancer patients who had undergone colorectal liver metastasectomy were included in the study. The mutant *KRAS* genotype was associated with CIP2A overexpression. CIP2A expression was an independent prognostic marker in patients with wild-type *KRAS* metastatic colorectal cancer after colorectal liver metastasectomy (relative risk = 1.873, *P* = 0.019). Targeted silencing of CIP2A in Caco-2 cells (wild-type *KRAS*) led to decreased expression of pERK/ERK and decreased cell proliferation. Overexpression of mutant KRAS G12D in Caco-2 cells led to an increase in CIP2A expression and cell proliferation. In Caco-2 cells with the KRAS G12D, KRAS overexpression preserved the regulation effect of CIP2A in KRAS and abrogated the impact of CIP2A regulation on pERK/ERK and cell proliferation. CIP2A inhibition also increased the efficacy of cetuximab in Caco-2 cells.

**Conclusions:**

CIP2A is an independent prognostic marker in patients with wild-type *KRAS* metastatic colorectal cancer after colorectal liver metastasectomy.

## Background

Approximately one-third of patients with colorectal cancer develop metastatic disease, with metastases commonly occurring in the liver [1,2]. The standard treatment for patients with colorectal liver metastasis is colorectal liver metastasectomy [2]; however, the rate of recurrence is high (~70%) for first-time colorectal liver metastasectomy patients [3-5]. Thus, the identification of novel oncogenes or targets as biomarkers for colorectal liver cancer recurrence is necessary.

**Figure 1:**
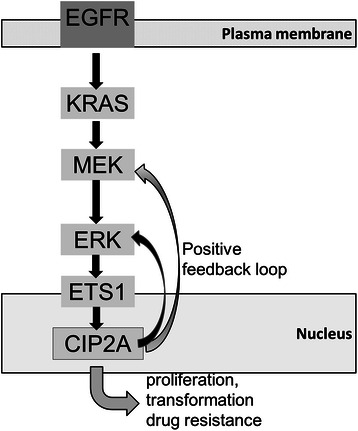
Model of CIP2A involvement in the EGFR-RAS signaling pathways. ETS1 mediates CIP2A overexpression in human cancers with increased EGFR-MEK-ERK pathway activity. A positive feedback loop of CIP2A and MEK/ERK signaling pathways is shown.

Cancerous inhibitor of protein phosphatase 2A (CIP2A) is a recently identified oncoprotein that is overexpressed in several malignancies, including leukemia, breast, gastric, prostate, lung, ovarian, head and neck carcinoma, and colorectal cancer [6-21]. CIP2A plays an important role in cell proliferation, transformation, drug resistance and maintenance of a malignant cellular phenotype [22]. Notably, CIP2A is associated with the epidermal growth factor receptor (EGFR) signaling pathway (Figure 1). CIP2A expression is positively correlated with EGFR expression and amplification in serous ovarian cancer [23]. Furthermore, in hepatoma cells, CIP2A expression is also associated with resistance to the anti-EGFR bio-agent, erlotinib (Tarceva), *in vitro* [24]. It is well documented that EGFR signaling is the most important pathway in treating metastatic colorectal cancer [25,26]. Therefore, the interplay between EGFR signaling and CIP2A in metastatic colorectal cancer warrants further investigation.

V-Ki-Ras2 Kirsten Rat Sarcoma Viral Oncogene (KRAS) is the most important downstream effector in the EGFR pathway. Approximately 40% of patients with metastatic colorectal cancer carry *KRAS* mutations affecting codons 12 and 13 in exon 2 [5]. Indeed, mutation of *KRAS* is a predictive marker of cetuximab efficacy in metastatic colorectal cancer patients [26,27]. However, to date, the prognostic value of *KRAS* mutations in metastatic colorectal cancer remains inconclusive [5,28-33]. Our previous study in colorectal cancer cell lines revealed that the *KRAS* G12D mutation decreased the impact of CIP2A on downstream effectors of the EGFR signaling pathway, when cells were treated with temsirolimus [34]. To the best of our knowledge, the interaction between *KRAS* mutant status and CIP2A has not been previously explored following colorectal liver metastasectomy.

In this study, we therefore investigated the association of CIP2A expression and *KRAS* genotype in the context of colorectal liver metastasectomy. We found that CIP2A only acts as an independent prognostic marker in patients with wild-type *KRAS* metastatic colorectal cancer after colorectal liver metastasectomy.

## Methods

### Patients and tissue blocks

A total of 220 patients undergoing colorectal liver metastasectomy at Taipei Veterans General Hospital in Taiwan, were enrolled in our study between January 2000 and January 2010. Disease stage was assessed based on the American Joint Committee on Cancer staging system, 6th edition. Clinicopathological staging and clinical course were determined by searching a computer database containing detailed information. The medical residual samples of patients came from residual sample bank of Taipei Veterans General Hospital and this study was approved by the Institutional Review Board of Taipei Veterans General Hospital (VGHIRB No 2012-03-027BC). Thus, The VGHIRB waive the requirement of inform consent form. The decision to perform hepatic resection was made by a multidisciplinary specialist committee. After hepatic resection, decisions regarding adjuvant chemotherapy were made on an individual patient basis at the discretion of the attending physicians. Patients were followed until the end of assessment (March 2012) or death, whichever occurred earlier. Patient follow-ups occurred at least every 3 months from the time of hepatic resection for the first 2 years, then every 6 months for the next 5 years, and subsequently annually until the patient’s death. Overall survival (OS) was defined as the period from liver surgery to death due to cancer.

### Immunohistochemistry (IHC)

CIP2A expression was assessed by IHC using monoclonal antibodies to CIP2A (NB100-74663, 1:1200; Novus Biologicals, Littleton, CO, USA). IHC staining was performed as previously described [10]. Negative (no primary antibody) and positive tissue controls (colon carcinoma) were stained in parallel with each set of tumor specimens studied.

IHC staining was evaluated by two pathologists who were unaware of the patients’ clinical information. The intensity of stained cells was scored as 0, 1, 2 or 3. Percentages of stained cells were counted, and a final immunohistochemical score (H-score) was calculated by summing the products of the staining intensities (0–3) and distributions (0–100%). H-scores ranged from 0–300. For CIP2A staining, an H-score of  ≥ 150 points was defined as strongly positive, whereas an H-score of < 150 points was defined as weakly positive.

### DNA extraction and *KRAS* mutation analyses

Tumor regions were macrodissected and examined to confirm that at least 80% of the cells in the tissue were cancer cells. DNA extraction was performed using a Nucleon HT DNA extraction kit (Piscataway, NJ, USA), according to the manufacturer’s instructions. Exon 2 of *KRAS* was separately amplified as previously described [35]. Purified PCR products were sequenced using the BigDyeR Terminator v3.1 cycle sequencing kit (Applied Biosystems, Foster City, CA, USA) and analyzed using a 3730 ABI capillary electrophoresis system (Applied Biosystems).

### Cell culture and transfection

The Caco-2 human colon cancer cell line, harboring wild-type *KRAS* (American Type Culture Collection; ATCC) was maintained in Dulbecco’s Modified Eagle Medium (Gibco, Grand Island, USA). Cells were maintained at 37°C in a humidified atmosphere of air and 5% CO_2_. Transfections were performed using Lipofectamine 2000 in accordance with the manufacturer’s protocol (Invitrogen, Massachusetts, USA). The *KRAS* wild type genotype in Caco-2 cells was confirmed by analysis of *KRAS* codon 12 and 13 mutations.

### Cell viability assay

Caco-2 cells were seeded in 96-well plates (10,000 cells/well) and after incubation for 12 h, cells were treated with cetuximab (Merck, Taiwan). Cell viability was then assessed using the TACS MTT cell proliferation assay kit (TREVIGEN, Gaithersburg, MD, USA), according to the manufacturer’s instructions. The dose–response or time-response curves were analyzed using GraphPad Software (Institute for Scientific Information, Philadelphia, PA, USA).

### Immunoblot analysis

Immunoblot analysis was performed as previously described [10]. The following proteins were evaluated by immunoblot: CIP2A (1:500; Novus Biologicals, CO, USA), pERK (1:1000; Cell Signaling Technology, Boston, MA, USA), ERK (1:5000; Zymed, Grand Island, NY, USA) and β-actin (1:500; Santa Cruz Biotechnology, Santa Cruz, CA, USA). β-actin was used as a loading control. Immunoblot quantification was performed using Image J software (http://rsb.info.nih.gov/ij/index.html).

### Knockdown of CIP2A and KRAS in colon cancer cells

The siRNA construct targeting CIP2A (pLKO.1-shCIP2A, TRCN0000135532, target sequence: (5′-CCACAGTTTAAGTGGTGGAAA-3′) and non-targeting siRNA control (pLKO.1-shLuc) were obtained from the National RNAi Core Facility, Taiwan (http://rnai.genmed.sinica.edu.tw/index). Lentivirus production and infection were performed as previously described [10]. The KRAS G12D mutant construct (pCMV6-Entry-KRAS G12D, RC400104) and control vector (pCMV6-AC-GFP, PS100010) were purchased from Origene (Rockville, Maryland, USA).

### Statistical and survival analyses

The correlations between clinicopathological variables and genomic alterations were analyzed by χ^2^ test or Fisher’s exact test. Survival was estimated using the Kaplan–Meier method, and the log-rank test was used for comparison of survival curves as well as for univariate analysis. The Cox proportional hazards model was applied for multivariate analyses. Variables with *P*-values ≤ 0.010 in the log-rank test were entered in multivariate analyses. The *t*-test was used to compare data from the proliferation studies. A two-sided *P-*value of < 0.05 was considered statistically significant. SPSS software (version 16.00, SPSS, Chicago, IL, USA) was used for all statistical analyses.

## Results

### Patient characteristics and association of clinical parameters with CIP2A expression

**Figure 2:**
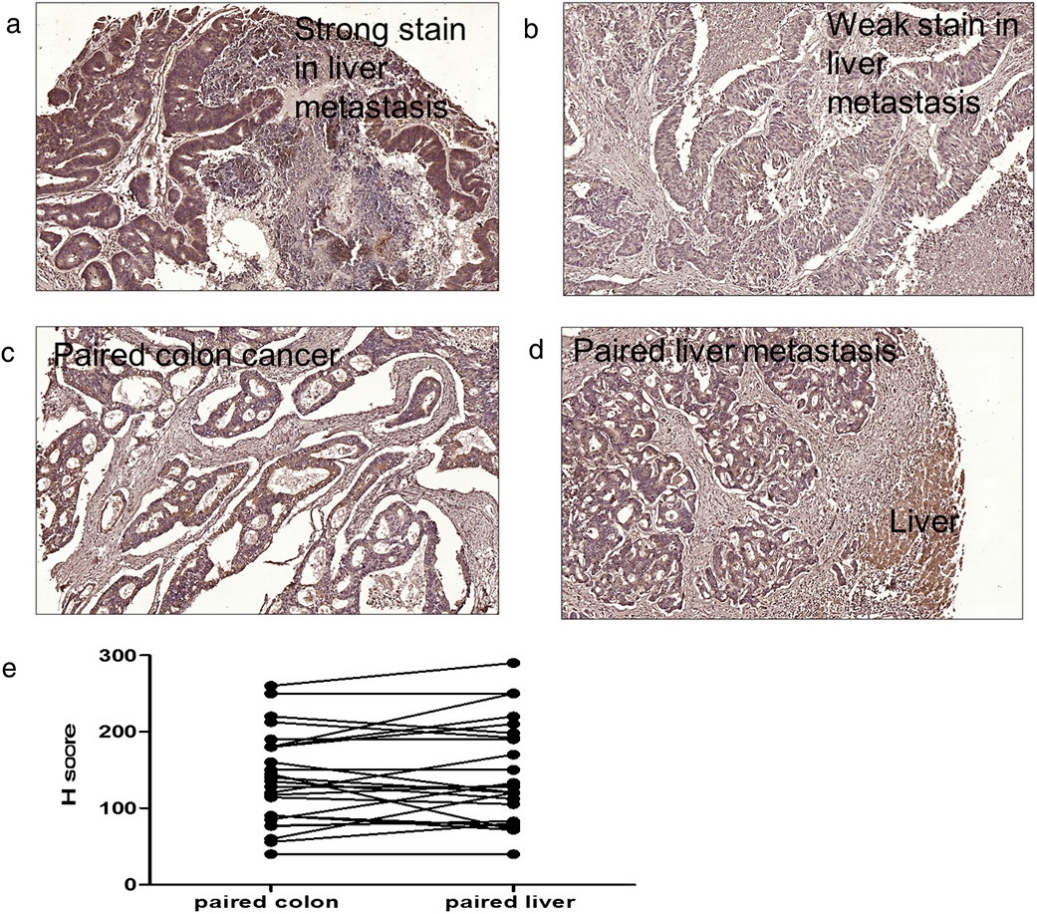
Immunohistochemical (IHC analysis of CIP2A expression in patients with colorectal cancer. Representative examples of CIP2A expression: **(a)** strong expression in colorectal liver metastasis, **(b)** weak expression in colorectal liver metastasis, **(c)** staining in paired colon cancer, **(d)** colorectal metastasis tissues, and **(e)** H-score in paired colon cancer and liver metastasis samples. CIP2A was not consistently overexpressed in colon cancer compared with colorectal liver metastasis in paired tissue specimens.

A total of 220 patients who had undergone colorectal liver metastasectomy were enrolled in our study (Table 1). The median age at diagnosis was 62.0 years (range: 30–87 years). The median OS after colorectal liver metastasectomy was 51.0 months, and the 5-year survival rate was 52.7%. To investigate the association between CIP2A expression and patient clinical parameters, CIP2A expression was examined in colorectal liver metastases sections by IHC staining (representative images are shown in Figure 2a and b). Ninety-one patients (41.4%) exhibited strong CIP2A expression. CIP2A expression was not significantly correlated with sex, age, initial stage at diagnosis, location of primary tumor, pathology, grade, margin, distribution of liver metastasis, number of liver metastases, size of liver metastasis or extrahepatic metastasis. However, CIP2A overexpression was associated with *KRAS* mutation status (*P* < 0.001). Among patients with *KRAS* codon 12 mutations, 57.6% exhibited strong CIP2A expression. Among patients with *KRAS* codon 13 mutations, 54.5% exhibited strong CIP2A expression. Only 30.5% of patients with the wild-type *KRAS* genotype exhibited strong CIP2A expression.

**Table 1:** **Association between clinicopathological parameters and CIP2A expression in patients after colorectal liver metastasectomy**

n = 220	CIP2A					
		Weak expression		Strong expression		
		n	(%)	n	(%)	*P-*value
Sex	Female	44	(53.7)	38	(46.3)	0.248
	Male	85	(61.6)	53	(38.4)	
Age (y/o)	≤65	80	(63.5)	46	(36.5)	0.090
	>65	49	(52.1)	45	(47.9)	
Initial stage at diagnosis	I-III	57	(44.2)	30	(33.0)	0.094
	IV	72	(55.8)	61	(67.0)	
Location	Colon	88	(57.5)	65	(42.5)	0.610
	Rectum	41	(61.2)	26	(38.8)	
Pathology	Adenocarcinoma	127	(59.6)	86	(40.4)	0.101
	Mucinous adenocarcinoma	2	(28.6)	5	(71.4)	
Grade	Low	121	(93.8)	90	(98.9)	0.060
	High	8	(6.2)	1	(1.1)	
Margin	R0-1	125	(96.9)	88	(96.7)	0.935
	R2	4	(3.1)	3	(3.3)	
Distribution	Unilateral	113	(59.8)	76	(40.2)	0.392
	Bilateral	16	(51.6)	15	(48.4)	
Number	≤3	101	(60.1)	67	(39.9)	0.422
	>3	28	(53.8)	24	(46.2)	
Size (cm)	≤5	107	(59.8)	72	(40.2)	0.473
	>5	22	(53.7)	19	(46.3)	
Extrahepatic metastasis	No	107	(82.9)	74	(81.3)	0.756
	Yes	22	(17.1)	17	(18.7)	
KRAS	Wild-type	91	(69.5)	40	(30.5)	<0.001
	Codon 12 mutation	28	(42.4)	38	(57.6)	
	G12D	13	(40.6)	19	(59.4)	
	G21V	7	(41.2)	10	(58.8)	
	G12C	5	(41.7)	7	(58.3)	
	G12R	1	(100.0)	0	(0.0)	
	G12S	2	(50.0)	2	(50.0)	
	Codon 13 mutation	10	(45.5)	12	(54.5)	
	G13D	9	(45.0)	11	(55.0)	
	G13C	0	(0.0)	1	(100.0)	
	G13V	1	(100.0)	0	(0.0)	
	Codon 14 mutation	0	(0.0)	1	(100.0)	
	V14I	0	(0.0)	1	(100.0)	

*Abbreviations*: *CIP2A* Cancerous inhibitor of protein phosphatase 2A, *KRAS* v-Ki-ras2 Kirsten rat sarcoma viral oncogene homolog.

The intensity of CIP2A staining in paired colon cancer and colorectal liver metastasis samples was also compared in 24 patients (representative images are shown in Figure 2c and d). CIP2A expression was similar between paired colon cancer and colorectal liver metastasis samples, with no significant difference in H-score between primary and metastatic tumors (Figure 2e).

### Prognostic factors for OS according to univariate and multivariate analyses in patients after colorectal liver metastasectomy

To clarify the role of CIP2A in patients following colorectal liver metastasectomy, the Cox proportional hazards model was applied (Table 2). In the univariate model, initial stage at diagnosis, number of colorectal liver metastases, margin and CIP2A expression (hazard ratio [HR] = 1.447, *P =* 0.049) were prognostic factors. However, in the multivariate model, CIP2A expression was not an independent prognostic factor after controlling for other risk factors (HR = 1.373, *P* = 0.096).

**Table 2:** **Prognostic factors for overall survival according to univariate and multivariate analyses in patients with both wild-type and mutant KRAS metastatic colorectal cancer after colorectal liver metastasectomy**

n = 220	Univariate		Multivariate	
Variable	Hazard ratios (95% CI)	*P-*value	Hazard ratios (95% CI)	*P-*value
Age > 65 (y/o)	1.055 (0.729–1.526)	0.778	—	—
Initial stage IV at diagnosis	1.668 (1.129–2.462)	0.010	1.336 (0.885–2.016)	0.168
Bilobar liver metastases	1.494 (0.902–2.475)	0.119	—	—
Size > 5 cm	1.268 (0.803–2.002)	0.309	—	—
Number > 3	1.932 (1.287–2.903)	0.002	1.753 (1.151–2.670)	0.009
High grade	1.385 (0.915–2.096)	0.123	—	—
Margin R2	3.112 (1.350–7.172)	0.008	2.087 (1.200–6.567)	0.017
Extrahepatic metastasis	1.462 (0.937–2.282)	0.094	1.255 (0.798–1.973)	0.326
CIP2A overexpression	1.447 (1.001–2.092)	0.049	1.373 (0.946–1.992)	0.096

*Abbreviations*: *CIP2A* Cancerous inhibitor of protein phosphatase 2A, *KRAS* v-Ki-ras2 Kirsten rat sarcoma viral oncogene homolog.

### Impact of CIP2A levels on OS in patients after colorectal liver metastasectomy according to *KRAS* genotype

**Figure 3:**
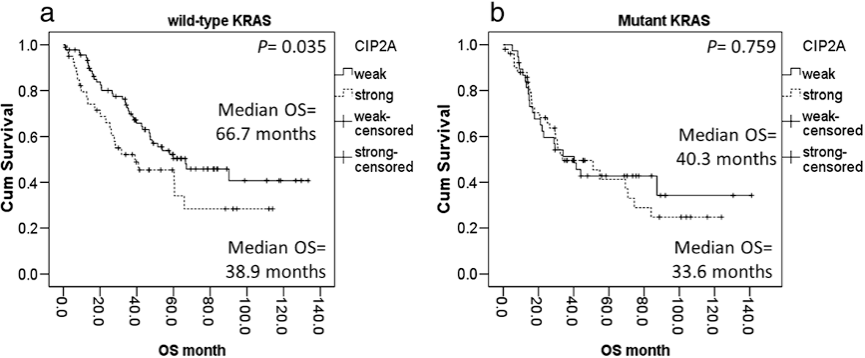
Kaplan–Meier survival plot of overall survival (OS) by *KRAS* genotype. **(a)** OS was significantly worse in patients with wild type *KRAS* and strong CIP2A expression, compared with patients with wild type *KRAS* and weak CIP2A expression (*P* = 0.035). **(b)** No difference in OS associated with CIP2A expression was observed in mutant *KRAS* patients (*P* = 0.759).

OS after colorectal liver metastasectomy was significantly worse in patients with strong CIP2A expression compared with those with weak CIP2A expression, in patients with wild-type *KRAS* (Figure 3a, *P* = 0.035). In contrast, we observed no difference in OS between strong and weak CIP2A expression in patients with mutant *KRAS* (Figure 3b, *P* = 0.759).

### Prognostic factors for OS according to univariate and multivariate analyses in patients with wild-type *KRAS* after colorectal liver metastasectomy

To clarify the role of CIP2A in patients exhibiting the wild-type *KRAS* genotype, the Cox proportional hazards model was applied (Table 3). In the univariate model, the number of colorectal liver metastases, grade, margin and CIP2A expression were prognostic factors, whereas age, initial stage at diagnosis, distribution, size of colorectal metastases and extrahepatic metastasis were not. Significant factors in the univariate model were subsequently used in the multivariate model. After controlling for other risk factors, CIP2A expression was still an independent prognostic factor in patients with wild-type *KRAS* genotype (HR = 2.109, *P* = 0.006).

**Table 3:** **Prognostic factors for overall survival according to univariate and multivariate analyses in patients with wild-type KRAS metastatic colorectal cancer after colorectal liver metastasectomy**

n = 131	Univariate		Multivariate	
Variable	Hazard ratios (95% CI)	*P-*value	Hazard ratios (95% CI)	*P-*value
Age > 65 (y/o)	1.045 (0.627–1.742)	0.865	—	—
Initial stage IV at diagnosis	1.660 (0.999–2.757)	0.050	1.445 (0.856–2.439)	0.168
Bilobar liver metastases	1.049 (0.477–2.308)	0.905	—	—
Size > 5 cm	0.865 (0.411–1.820)	0.703	—	—
Number > 3	2.045 (1.167–3.586)	0.013	2.084 (1.200–3.621)	0.009
High grade	1.863 (1.032–3.362)	0.039	2.031 (1.101–3.744)	0.023
Margin R2	3.559 (1.092–11.600)	0.035	3.701 (1.111–12.330)	0.033
Extrahepatic metastasis	1.472 (0.782–2.771)	0.231	—	—
CIP2A over-expression	1.751 (1.041–2.946)	0.035	2.109 (1.236–3.600)	0.006

*Abbreviations*: *CIP2A* Cancerous inhibitor of protein phosphatase 2A, *KRAS* v-Ki-ras2 Kirsten rat sarcoma viral oncogene homolog.

### Association of CIP2A expression, *KRAS* genotype, cetuximab and proliferation in wild-type *KRAS* Caco-2 colon cancer cells

**Figure 4:**
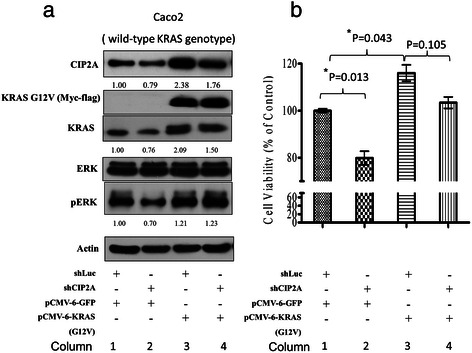
Interaction between CIP2A, *KRAS* genotype and proliferation in the Caco-2 *KRAS* wild-type cell line. The result of immunoblot and proliferation assay is shown in **(a)** and **(b)**, respectively. Column 1 *vs.* 2: CIP2A knockdown by shCIP2A resulted in decreased CIP2A, KRAS, and pERK expression as well as decreased proliferation; Column 1 *vs.* 3: KRAS overexpression by pCMV6-KRAS G12D resulted in increased KRAS, CIP2A, and pERK expression, as well as increased proliferation; Column 3 *vs.* 4: in Caco-2 cells with KRAS overexpression by pCMV6-KRAS G12D, knockdown of CIP2A by shCIP2A resulted in decreased CIP2A and KRAS expression. However, it did not cause significantly decreased pERK expression or decreased proliferation (*P < 0.05).

**Figure 5:**
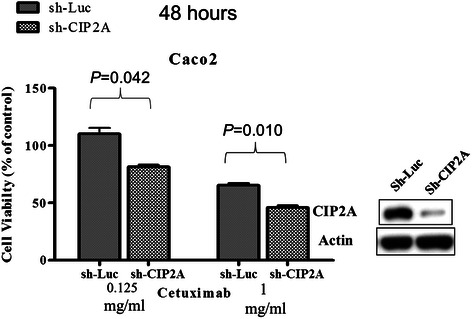
Silencing of CIP2A in Caco-2 cells leads to decreased resistance to cetuximab. Immunoblot analysis of CIP2A expression in control (shLuc) and CIP2A knockdown (shCIP2A) cells.

To explain our clinical findings, we conducted additional studies in Caco-2 colon cancer cells, which express wild-type *KRAS*. Targeted silencing of CIP2A using shCIP2A led to decreased expression of CIP2A, KRAS, and pERK (Figure 4a), and a concomitant decrease in cell proliferation (Figure 4b, *P* = 0.013). Overexpression of mutant KRAS (pCMV6-KRAS G12D, Figure 4a) led to increased expression of KRAS, CIP2A, and pERK, and increased cell proliferation (Figure 4b, *P* = 0.043). While targeted silencing of CIP2A also led to decreased CIP2A expression in cells transfected with pCMV6-KRAS G12D (Figure 4a), we did not observe a decrease in the expression of pERK/ERK or a significant decrease in proliferation. Last, the efficacy of cetuximab in Caco-2 cells increased after CIP2A knockdown compared with control (Figure 5).

## Discussion

To the best of our knowledge, this is the first study to report an association between CIP2A expression and *KRAS* genotype in patients with metastatic colorectal cancer after colorectal liver metastasectomy. After adjusting for other confounding factors, we found that CIP2A acts as an independent prognostic marker in patients with wild-type *KRAS* metastatic colorectal cancer after colorectal liver metastasectomy.

Overexpression of CIP2A is associated with tumor aggressiveness, lymph node and lymphovascular involvement, and advanced stage colon cancer, which may partially explain why CIP2A functions as a prognostic marker in patients with wild-type *KRAS* metastatic colorectal cancer after colorectal liver metastasectomy [10,36]. CIP2A overexpression is also associated with colon cancer cell proliferation, tumorigenesis *in vitro*, and resistance to cetuximab, 5-fluorouracil, oxaliplatin and SN38 (an active metabolite of irinotecan) [10,34]. Cetuximab, 5-fluorouracil, oxaliplatin and irinotecan are commonly used for post-operative chemotherapy after colorectal liver metastasectomy or for salvage chemotherapy in the treatment of metastatic colorectal cancer [3].

In our analysis, the value of CIP2A as a prognostic marker was limited to patients with wild-type *KRAS* metastatic colorectal cancer after colorectal liver metastasectomy. This observation may be explained by multiple previous reports. First, Zhao *et al.* investigated *Helicobacter pylori* infection-induced CIP2A expression, and determined that it was dependent on RAS/mitogen-activated protein kinase (MAPK)/extracellular signal-regulated kinase (ERK) pathways, indicating that EGFR pathway activation increased CIP2A expression [37]. Khanna *et al.* [38] further concluded that CIP2A overexpression was dependent on the EGFR-ERK-ETS1 signaling pathway. Both studies illustrate that the EGFR pathway interacts with CIP2A *in vitro* and that the interaction is bi-directional. Furthermore, studies by Bockelman *et al.* demonstrated that EGFR protein expression and amplification were associated with CIP2A overexpression *in vivo* [23]. Similarly, we observed that a mutant *KRAS* genotype was associated with CIP2A overexpression. Finally, in colorectal cancer, the EGFR/RAS pathway is an important signaling pathway. KRAS is well established as an important downstream effector of the EGFR signaling pathway, and mutational activation of *KRAS* by further active downstream effectors like ERK induces drug resistance to EGFR antagonists (for example, cetuximab) [39]. The results of our cellular experiments support this finding. Indeed, we observed that targeted silencing of CIP2A in Caco-2 cells expressing mutant KRAS G12D led to decreased expression of CIP2A expression but not pERK/ERK. Overexpression of mutant KRAS G12D impaired the suppression of pERK/ERK and effects on cell proliferation mediated by CIP2A silencing. Based on the above results, CIP2A acts as a prognostic marker in patients with wild-type *KRAS* metastatic colorectal cancer following colorectal liver metastasectomy, because the mutational activation of KRAS weakens CIP2A regulation on cell survival.

One constraint of our study is that a limited number of patients received cetuximab (EGFR antagonist) as a bio-chemotherapy throughout the entire course of treatment. Thus, we could not demonstrate that CIP2A expression is a predictive marker of response to cetuximab in metastatic colorectal cancer patients with wild-type *KRAS*. Further studies are therefore required to investigate this relationship.

## Conclusions

Mutant *KRAS* is associated with CIP2A overexpression. CIP2A is an independent prognostic marker in patients with metastatic colorectal cancer exhibiting wild-type *KRAS* after colorectal liver metastasectomy.

## Abbreviations

CIP2A: Cancerous inhibitor of protein phosphatase 2A
EGFR: Epidermal growth factor receptor
ETS1: v-ets avian erythroblastosis virus E26 oncogene homolog 1
KRAS: v-Ki-Ras2 Kirsten Rat Sarcoma Viral Oncogene
IHC: Immunohistochemistry
MAPK: Mitogen-activated protein kinase
ERK: Extracellular signal-regulated kinase
